# Caloric restriction: implications for sarcopenia and potential mechanisms

**DOI:** 10.18632/aging.103987

**Published:** 2020-11-21

**Authors:** Wen-Qing Xie, Wen-Feng Xiao, Kun Tang, Yu-Xiang Wu, Pei-Wu Hu, Yu-Sheng Li, Yu Duan, Shan Lv

**Affiliations:** 1 Deparment of Orthopedics, Xiangya Hospital, Central South University, Changsha 410008, Hunan, China; 2 National Clinical Research Center for Geriatric Disorders, Xiangya Hospital, Central South University, Changsha 410008, Hunan, China; 3 Discipline Construction Office, Xiangya Hospital, Central South University, Changsha 410008, Hunan, China; 4 School of Kinesiology, Jianghan University, Wuhan 430056, China; 5 Department of Scientific Research, Xiangya Hospital, Central South University, Changsha 410008, Hunan, China; 6 Department of Geriatric Endocrinology, The First Affiliated Hospital of Nanjing Medical University, Nanjing 210029, Jiangsu, China

**Keywords:** aging, calorie restriction, sarcopenia, cellular mechanism

## Abstract

Sarcopenia is a potential risk factor for weakness, disability and death in elderly individuals. Therefore, seeking effective methods to delay and treat sarcopenia and to improve the quality of life of elderly individuals is a trending topic in geriatrics. Caloric restriction (CR) is currently recognized as an effective means to extend the lifespan and delay the decline in organ function caused by aging. In this review, we describe the effects of CR on improving muscle protein synthesis, delaying muscle atrophy, regulating muscle mitochondrial function, maintaining muscle strength, promoting muscle stem cell (MuSC) regeneration and differentiation, and thus protecting against sarcopenia. We also summarize the possible cellular mechanisms by which CR delays sarcopenia. CR can delay sarcopenia by reducing the generation of oxygen free radicals, reducing oxidative stress damage, enhancing mitochondrial function, improving protein homeostasis, reducing iron overload, increasing autophagy and apoptosis, and reducing inflammation. However, the relationships between CR and genetics, sex, animal strain, regimen duration and energy intake level are complex. Therefore, further study of the proper timing and application method of CR to prevent sarcopenia is highly important for the aging population.

Sarcopenia is a syndrome characterized by progressive and generalized loss of skeletal muscle mass and strength and, according to the European Working Group on Sarcopenia in Older People (EWGSOP), is accompanied by a risk of adverse outcomes such as physical disability, poor quality of life and death [1]. As the population has aged, sarcopenia, which is a potential risk factor for weakness, disability and death in elderly individuals, has become an important topic [2]. Caloric restriction (CR), a regimen in which caloric intake is reduced by 20-50% without causing malnutrition, is a classical antiaging intervention [3, 4]. Therefore, research to determine whether CR can delay muscle aging is important for the prevention and treatment of sarcopenia in elderly individuals. In this article, the related research on this topic and the possible mechanism through which CR delays sarcopenia are reviewed.

## Summary ot the current sarcopenia situation

Epidemiological investigations have indicated that the muscle mass of the human body decreases by approximately 1.5% yearly after the age of 50 and by 2.5-3.0% yearly after the age of 60 [5, 6]. The incidence rate of sarcopenia among individuals over 80 years old is as high as 50% [7]. Studies have shown that a 10% decrease in muscle mass leads to a decrease in immune function and an increase in the risk of infection. A 20% reduction in muscle mass results in muscle weakness, a decreased ability to participate in activities of daily living, and an increased risk of falling. A 30% reduction in muscle mass results in disability, loss of independent living ability, and failure of wound and pressure ulcer healing. A 40% reduction in muscle mass results in a markedly increased risk of death from pneumonia, respiratory dysfunction, etc. [8]. In addition, muscle is a protein repository and the primary tissue site of glycolipid metabolism. Muscle is responsible for the consumption of nearly 80% of the body’s glucose content, and its resting metabolic rate accounts for 30% of the entire body’s resting metabolic rate [9].

The main manifestations of sarcopenia in elderly individuals are a decreased cross-sectional area of muscle fibers and reduced muscle strength and function [10]. Clinical studies have shown that the reduction in muscle mass is much greater in the lower limbs than in the upper limbs [8]. Gait speed or the short physical performance battery (SPPB) are commonly used to assess muscle function [11]. Muscle strength tends to decrease with age, as manifested by reduced grip strength and knee joint extension, weakened hip joint bending activity, decreased pace, and increased time to maximal muscle contraction compared with those of young individuals [12]. Additionally, the number and the proliferation and differentiation abilities of muscle stem cells (MuSCs), which play an important role in muscle cell regeneration, are reduced. The number of MuSCs in aged mice is 50% lower than that in young mice [13].

Currently, the treatment methods for sarcopenia remain limited to improving nutrition and enhancing exercise. Seeking effective methods to delay and treat sarcopenia, thus improving the quality and prolonging the life of elderly individuals, has become a trending research topic in modern geriatric medicine.

## Effect of caloric restriction on sarcopenia

**Figure 1:**
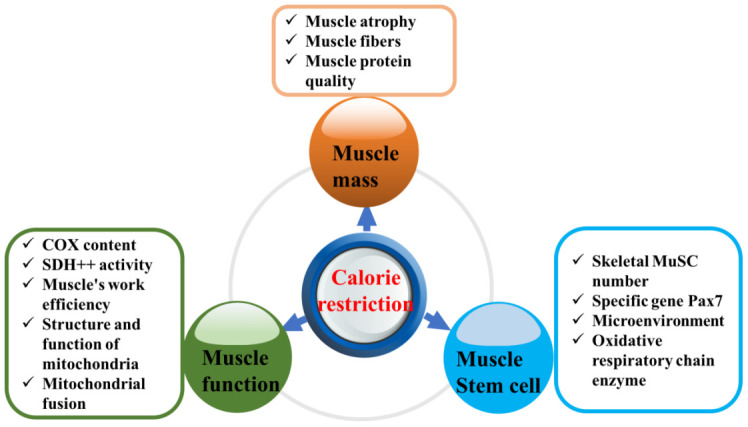
**Effect of calorie restriction on sarcopenia.**

CR has recently been found to not only extend the lifespan but also reduce or delay the occurrence of aging-related diseases [14]. Organisms in CR experimental groups have shown less pathological damage, lower disease risk during aging, longer lifespans and better maintenance of metabolic health than those in the corresponding control groups. For example, in yeast, reducing the glucose content in the culture medium from 2% to 0.5% significantly extended both the chronological and replicative lifespans [15]. The above conclusions have been confirmed in early studies of relatively simple model organisms (including yeast, nematodes, fruit flies, etc.), and subsequent studies have shown that dietary restriction is also effective in mammals such as mice and primates [3, 16–18]. More interestingly, research on nematodes published in Cell in 2014 revealed that this benefit could not only be realized in the present generation but also be passed on to future generations [19]. In addition, the risk of sarcopenia, type 2 diabetes, cancer and cardiovascular diseases was significantly decreased in rhesus monkeys in the CR group compared with those in the control group [20]. Can CR improve sarcopenia in elderly individuals? This review elaborates on this issue based on the aspects shown in Figure 1.

### Caloric restriction maintains muscle mass and muscle homeostasis

Muscle fibers can be divided into three types according to their metabolic energy conversion rate: fatigue-resistant slow-twitch type I muscle fibers, fast-twitch type IIa fibers and type IIb, or “intermediate” fibers [21]. Muscle protein synthesis decreases and the muscle protein degradation rate increases with age. Studies have shown that with aging, the levels of hormones related to anabolism (e.g., testosterone, growth hormone, and insulin-like growth factor 1 (IGF-1)) decrease and the activities of enzymes related to protein decomposition (e.g., ubiquitin proteases, cathepsin and calcium activators) increase [22]. CR has a protective effect on muscular atrophy in rodents and primate mammals, and the number of satellite cells and the content of collagen VI were found to increase after short-term 35% CR or 50% CR in 17-month-old rats [23]. Treatment with resveratrol, which mimics CR, was confirmed to prevent muscle loss and decreases in the size of myofibers, improve grip strength and abolish excessive fat accumulation in aged rats. *In vitro*, resveratrol inhibited the palmitate acid-mediated reductions in the myosin heavy chain content and myotube diameter [24]. Time-restricted eating (TRE), a regimen in which all daily calories are consumed within a truncated period of time, is another regimen of CR. A recent report indicated equivalent lean mass accretion and increases in skeletal muscle thickness in the group subjected to daily TRE, in which all calories were consumed within an average of ~7.5 h/day and a control group, in which calories were consumed within an average of ~13 h/day [25].

Analysis of lateral thigh muscle slices showed that rats subjected to 50% CR retained a number and composition of fibers similar to that of rats in the control group and rats subjected to 35% CR by a mechanism possibly related to a reduction in inflammation [26]. Yang et al. found through further research that muscle protein quality may be improved by long-term CR through enhancement of autophagy and a reduction in inflammation to maintain muscle homeostasis [4]. The most recent research in rhesus monkeys showed that CR induced profound changes in muscle composition and the cellular metabolic environment. At the tissue level, CR maintained the contractile content and attenuated age-related metabolic shifts among individual fiber types, accompanied by increased mitochondrial activity, altered redox metabolism, and reduced lipid droplet size [27].

### Caloric restriction improves muscle function

The muscle is an important motor organ that can guide the lever-like movements of the skeleton and enable the body to perform activities by shortening, elongating and contracting muscle fibers. Muscle function depends not only on the structure of the muscle tissue itself but also on ATP consumption to supply energy [28]. Mitochondria are the main sites of oxidative phosphorylation and ATP generation. Via intake of energy substrates (such as proteins, carbohydrates, and lipids), mitochondrial oxidative phosphorylation and ATP generation can be a source for the energy production, maintenance and metabolism required for athletic activities. Previous studies have indicated that skeletal muscle contraction accounts for most of the body’s total energy consumption [29]. Compared with 4-month-old mice, 30-month-old mice showed differences in the transcription and translation of up to 35 proteins, especially those related to redox homeostasis and iron loading [30]. Proteins participate in redox homeostasis; thus, mitochondria in aging muscle cells produce more free radicals than young muscle cells via cellular respiration. Recently, a study on cell metabolism showed that CR can extend the lifespan by regulating mitochondrial networks and promoting peroxisome production [31]. Elderly mice either lack cytochrome C oxidase (COX) activity or exhibit increased succinate dehydrogenase (SDH++) activity. However, the COX content was decreased and the activity of SDH++ was significantly decreased in the muscle of mice in the 50% CR group compared with the muscle of mice in the 35% CR and control groups [26]. Moreover, Almundarij et al. found that after caloric intake was reduced by 50% in rats, the work efficiency of muscle increased comparably, i.e., by 50%. Less energy was consumed to complete the same amount of work [32]. This effect may be due to the reduction in sympathetic nerve excitation and the conversion of endocrine hormones, thus increasing the energy supply available for muscle exercise and reducing the energy supply produced by heat. Limiting caloric intake can also significantly improve mitochondrial structure and function, increase the number of mitochondria and promote mitochondrial fusion, thus augmenting ATP synthesis and maintaining the body’s energy supply under starvation conditions.

### Caloric restriction improves the regeneration ability and microenvironment of muscle stem cells

MuSCs are one type of adult stem cell and are the most important participants in muscle regeneration [33]. In the neonatal period, MuSCs are highly activated and proliferate rapidly to support the rapid growth of the body. The number of MuSCs is the highest during this stage, accounting for approximately 30% of the total number of muscle nuclei. In adulthood, the number of MuSCs decreases, and the MuSCs present are usually in a resting state [34]. When muscle is damaged, the MuSCs in the resting state are activated, enter the proliferation stage and further differentiate and fuse to form myotubes, which exhibit an orderly arrangement and fuse to generate muscle fibers, thus forming muscles [35]. The number and the proliferation and differentiation abilities of MuSCs gradually decrease with increasing age.

A recent study found that CR can improve the function of adult stem cells, including the regeneration ability of skeletal MuSCs. To study whether CR can affect the rhythmic activity of stem cells during aging, researchers conducted a 25-week comparative observation of aged mice (60 weeks) that consumed a control diet or a diet with 30% fewer calories than the control diet. In this study [36], except for the reduction in body weight, the aging characteristics related to epidermal and muscle tissue in mice were significantly ameliorated in the CR group compared with the control group; for example, the capsule cuticle thickness was decreased, the fur thickness was increased, and the number of skeletal MuSCs increased. Even more surprisingly, genes involved in inflammation or mitochondrial DNA (mtDNA) repair were not regularly transcribed in the aging skeletal MuSCs of mice in the CR group, while genes related to cell self-balance were regularly transcribed. In other words, CR restores the aging stem cells of mice to a state similar to that observed in young stem cells, thus delaying aging.

Additional studies have indicated that not stem cells themselves but the stem cell microenvironment is the key factor mediating stem cell activation, proliferation and differentiation [37]. After short-term CR, the Notch signaling pathway can be activated in cells in the microenvironment, leading to increased expression of the stem cell-specific gene Pax7 in skeletal muscle and concurrent increases in mitochondrial number, oxidative respiratory chain enzyme expression and aerobic utilization rate in stem cells [38]. However, recent studies have reached different conclusions. Although CR increases the activity of stem cells, it leads to a delayed regeneration response to injury [13].

### Possible mechanism by which caloric restriction delays sarcopenia

**Figure 2:**
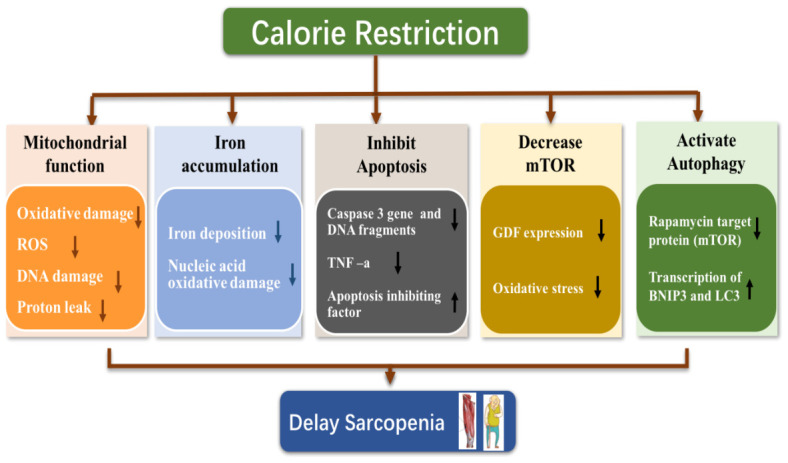
**Possible mechanism of calorie restriction delaying sarcopenia.**

CR delays sarcopenia via a complex mechanism. Current research generally focuses on the regulation of oxidative stress, mitochondrial function, inflammation, apoptosis and autophagy, as shown in Figure 2 [39–43].

### Caloric restriction alleviates the decline in mitochondrial function in skeletal muscle and decreases oxidative stress

Mitochondrial dysfunction is an important factor leading to age-related muscular atrophy [44]. Considering the dependence of skeletal muscle on ATP, loss of mitochondrial function, which can lead to a decrease in strength and endurance, is especially obvious in skeletal muscle. Previous experimental evidence showed that the oxidative phosphorylation ability of muscle mitochondria decreases with age. Notably, point mutations in mtDNA in aging muscles accumulate continuously, resulting in electron transfer chain abnormalities and fiber atrophy [45]. MtDNA is particularly vulnerable to oxidative damage because it is localized near the electron transport chain (ETC) and lacks protective tissue proteins. In addition, with aging, the efficiency of the primary mechanism for removing small mtDNA bases (base excision repair, BER) *in vivo* decreases variably. Moreover, the content of mitochondrial reactive oxygen species (ROS) increases with muscle aging, which may be the main cause of mtDNA mutations [46]. Studies on different species have found that the mtDNA damage associated with sarcopenia occurs mainly in genes encoding components related to mitochondrial respiratory complexes I and IV, mitochondrial ETC and other processes [47]. Aging-related mtDNA deletion mutations in fibers usually manifest as morphological distortions, including segmental atrophy, fiber division and breakage. Importantly, CR can reduce and prevent mtDNA deletion mutations and increase the ETC protein content [48].

CR can preserve the integrity and function of mitochondrial structure via reducing oxidative damage. Previous studies have shown that CR reduces proton leakage and ROS generation in mitochondria in skeletal muscle while enhancing the expression of ROS scavenging-related genes. In addition, CR may alter the fatty acid composition of the mitochondrial membrane, reduce lipid oxidation and reduce proton leakage [46]. Lass et al. found that CR can also reverse the generation of superoxide anion radicals, lipid peroxidation and mitochondrial protein damage related to age in skeletal muscle [49]. Moreover, Drew et al. reported that CR can reduce oxidative damage to mtDNA in the gastrocnemius muscle [50]. Interestingly, in a recent population study, CR of 25% for 6 months not only increased the synthesis of skeletal muscle mitochondria but also reduced DNA damage in middle-aged, overweight healthy individuals [51]. The increase in the mitochondrial number can be explained as a positive adaptation promoted by CR, because a larger number of mitochondria reduces the workload per mitochondria, thus limiting oxidative radical generation.

### Caloric restriction inhibits iron accumulation in skeletal muscle

Iron is an essential metal in the body and plays an important role in cellular biological activities, including oxygen and electron transport, drug metabolism, steroid and DNA biosynthesis, and other activities. Seventy percent of the iron in the body is stored in hemoglobin, and most remaining iron is stored in iron-containing proteins in the liver and in myoglobin in muscle cells [52]. Accumulating evidence shows that age-related iron overload underlies the pathogenesis of degenerative diseases, including Alzheimer's disease, Parkinson's disease, and sarcopenia, [53, 54].

Iron is an active metal with a high redox potential that can convert oxidant intermediates such as hydrogen peroxide to harmful oxygen free radicals. In addition, iron has been shown to catalyze the nitration of tyrosine residues, resulting in protein damage [55]. Excess iron can promote oxidative damage-mediated muscle deterioration, leading to muscle atrophy. Hofer et al confirmed the existence of iron overload in atrophic muscle [56]. In addition, studies have shown that the iron content increases with age in skeletal muscle mitochondria; moreover, addition of the iron chelator desferrioxamine was shown to delay oxidative damage and muscle atrophy in rats [54]. Notably, iron is more likely to accumulate under the sarcolemma than in muscle and commonly causes mtRNA oxidation, which increases the sensitivity of mitochondrial permeability transition pore (mPTP) opening, a process associated with apoptosis [57].

CR has been shown to reduce iron accumulation and oxidative damage in the kidneys of aged rats [58]. Recently, age-related iron accumulation in the gastrocnemius muscle was reported, and CR was found to ameliorate iron accumulation and oxidative damage to nucleic acids in mice [59]. Interestingly, inhibition of iron deposition by CR was positively correlated with forelimb grip strength, suggesting that iron accumulation in the muscles of elderly individuals may lead to loss of function. Therefore, the decrease in iron overload by CR may be an important direction for sarcopenia intervention.

### Caloric restriction inhibits skeletal muscle apoptosis

Accumulating evidence suggests that apoptosis may constitute a fundamental mechanism driving the onset and progression of sarcopenia [60]. Apoptosis is a process that ultimately leads to DNA fragmentation through specific signaling pathways, including nuclear DNA rotation, nuclear condensation, proteolysis, submembrane and cell division, apoptotic body formation, and phagocytosis by macrophages. There are two main pathways of apoptosis: activation of the apoptotic enzyme caspase through extracellular signaling and activation of caspases through the release of mitochondrial apoptosis activators [61]. These activated caspases can degrade important proteins in cells and induce apoptosis [62].

The gene expression and cleavage of pre-caspase-3 in the gastrocnemius muscle were significantly reduced in CR mice compared with control mice [63]. In addition, CR increased the content of apoptosis inhibitors in the cytoplasm. Recently, CR was also found to inhibit death receptor-induced myocyte apoptosis initiated by TNF-a in aged rats [64]. Further studies confirmed that the inhibitory effect of CR was achieved by blocking caspase-8 expression downstream of TNF-a [65].

### Caloric restriction suppresses mammalian target of rapamycin (mtor) signaling

Experimental data strongly suggest that mTOR activity increases during aging, beginning in middle age and resulting in progressively altered mitochondria, in turn leading to mitochondrial oxidative stress and thus the induction of catabolic processes, including protein degradation, apoptosis, and necrosis [66]. This elevated catabolic activity results in muscle fiber loss, atrophy, and damage [67]. Therefore, mTOR inhibition may delay the progression of sarcopenia by modulating multiple age-associated pathways.

A decade ago, rapamycin, a mTORC1 inhibitor, was reported to extend the lifespan of mice [68]. Rapamycin’s effect on aging skeletal muscle, however, was not explored until recently. Inhibition of mTOR with rapamycin prevents age-related muscle loss [69]. For example, rapamycin blocks GDF expression in elderly mice and prevents age-related muscle fiber loss [70]. At the cellular level, rapamycin decreases the number of myocytes with abnormal desmin accumulation and decreases the amount of desmin in muscle tissue. Recent evidence has shown that CR downregulates mTORC1 signaling in skeletal muscle independent of dietary protein intake [71]. Moreover, a paper published in 2019 indicated that the effects of CR on mTOR signaling in skeletal muscles are age-dependent [72]. CR altered mTOR signaling in the soleus muscles in middle-aged rats but not in young and adult rats.

### Caloric restriction activates autophagy

Autophagy is essential for overall cellular health because in some residual tissues, the lack of an autophagic response gradually results in the accumulation of damage within the cells, eventually leading to cell death and loss of tissue function. Thus, in both worm and fly studies, the proper initiation and execution of autophagy has been found to be associated with increased longevity [73].

The effect of autophagy on lifespan and cellular homeostasis is widely supported. Studies on the skeletal muscle cell line C2C12 showed that nutrient restriction leads to autophagy, which may be driven by at least two signaling pathways: Class III phosphoinositide 3-kinase-Beclin1 complex formation and reduced mTOR synthesis [74]. Another study showed that mTOR deficiency may extend the lifespan of worms by increasing autophagy [75]. Moreover, *in vivo* studies have demonstrated that CR can increase autophagic responses in skeletal muscle [76]. Additional studies have shown that CR regulates the transcription factor Forkhead box O3 (FOXO3), which is associated with human longevity [77], and recent studies have shown that muscle atrophy is associated with the expression of the transcription factor FOXO3 and other downstream target skeletal muscle atrophy-related proteins, namely, Atrogin 1 and MuRF1 [78]. Mammucari et al. [79] found that the autophagic process driven by FOXO3 transcription factors in skeletal muscle cells can ultimately lead to autophagic proteolysis by deactivating or rapidly inducing the transcription of BNIP3 and LC3.

### Controversies regarding the delay of sarcopenia by caloric restriction

Although some evidence indicates that CR reduces or delays many of the age-related defects that occur in rodent skeletal muscle, the levels of nutrition, different contents of dietary macronutrients, different timing and different animal strains used in these studies have potentially important effects on the observed effects of CR [80]. Currently, no consensus has been reached regarding the use of micronutrient supplementation in CR studies, and protocols vary widely [81]. Boldrin et al investigated the effect of short-term (2.5 months) and longer-term (8.5 and 18.5 months) CR on skeletal muscle in male and female C57BL/6 and DBA/2 mice [23]. Overall, CR extended the lifespan of C57BL/6 mice but not DBA/2 mice. The changes induced by CR did not persist with time and were independent of the dissimilarities between the two mouse strains. Additionally, short-term CR increased the number of satellite cells and collagen VI content in muscle but resulted in a delayed regenerative response to injury. Consistent with this finding, the *in vitro* proliferation of satellite cells derived from these muscles was reduced by CR. In addition, Mitchell et al reported that the interaction and contribution of each of these factors may impact the overall energetic balance of the organism, which determines the outcome of the intervention on health [82]. In contrast, in a study by Park et al., CR was found to promote muscle loss in aged mice [83]. Moreover, studies have shown that overweight and even obese elderly individuals live longer than normal-weight individuals. The survival times of overweight and obese individuals were longer than those of normal-weight individuals (2.3 years for men and 4.6 years for women) [84]. In addition, both men and women with overweight (BMI 25-30 kg/m^2^) had the longest disability-free life span [85]. Hence, CR should be used with caution in elderly adults, as excessive CR promotes muscle loss and a low body mass index and increases the risk of disability and mortality in elderly individuals.

## CONCLUSIONS

Sarcopenia is a prominent manifestation of human weakness and aging, presenting as the loss of skeletal muscle mass and strength with aging. Delaying aging while promoting healthy aging has historically been a challenge for humans. The protective effects of CR on sarcopenia are manifested as improved protein quality, maintenance of muscle strength, and enhanced muscle function, and these effects may be achieved via mitigation of cellular oxidative stress, promotion of mitochondrial function, alleviation of the inflammatory response, inhibition of apoptosis and activation of autophagy, and other mechanisms.

However, the relationships between CR and genetics, sex, animal strain, regimen duration and energy intake level are complex. From a translational therapeutic perspective, determining the proper timing and application method of CR to prevent sarcopenia in humans, especially in elderly adults, is challenging, as CR exacerbates weight loss in elderly individuals. Therefore, further study on CR and sarcopenia is highly important for the aging population.
